# Effect of Heat Treatment on Elastic Properties and Fracture Toughness of Fused Filament Fabricated PEEK for Biomedical Applications

**DOI:** 10.3390/polym14245521

**Published:** 2022-12-16

**Authors:** Ilia Vindokurov, Yulia Pirogova, Mikhail Tashkinov, Vadim V. Silberschmidt

**Affiliations:** 1 Laboratory of Mechanics of Biocompatible Materials and Devices, Perm National Research Polytechnic University, Komsomolsky Ave., 29, Perm 614000, Russia; 2 Wolfson School of Mechanical, Electrical and Manufacturing Engineering, Loughborough University, Loughborough LE11 3TU, UK

**Keywords:** biocompatible polymers, PEEK, mechanical properties, manufacturing parameters, heat treatment, infill orientation, fracture toughness

## Abstract

This work presents the results of an experimental investigation of the mechanical properties of polyetheretherketone (PEEK) specimens additively manufactured (AM) by using fused filament fabrication with different printing parameters and subjected to postprocessing heat treatment. Standard and compact tension samples were manufactured with a different infill angle using 0.4 mm and 0.6 mm nozzle diameters. Some of the samples were subjected to heat treatment at 220 °C after manufacturing. Tensile tests were conducted to determine the values of elastic modulus, tensile strength, as well as mode-I fracture toughness and critical strain energy release rate. Tensile properties of single-thread and as-delivered filaments were also studied. It was concluded that heat treatment significantly improved the elastic properties, tensile strength and fracture toughness of the AM PEEK samples: the fracture resistance increased by 33 to 45% depending on the stacking order, while the tensile strength increased by some 45–65%, with the elasticity modulus grown by up to 20%. Strain fields induced in specimens by crack propagation were captured with a digital image correlation technique and compared with results of numerical simulations implemented with the extended finite-element method (XFEM). Conclusions on the optimal parameters of 3D printing of PEEK were made.

## 1. Introduction

The fast development of new advanced polymeric materials in recent decades has resulted in the successful substitution of metals in structures with their plastic counterparts. Additive manufacturing (AM) proved to be an effective technology for the production of parts and structures with complex geometries and tailored behaviors. Compared to other established technologies, AM provides great flexibility for three-dimensional design of the material’s architecture based on digital models. One of the most affordable and commonly used AM techniques is fused filament fabrication (FFF) (also known as fused deposition modeling, FDM) [1,2,3,4,5,6,7,8,9]. During the FFF manufacturing process, a thermoplastic filament is fed into a heating nozzle and then—fused or liquefied—extruded and deposited on a surface according to a predetermined toolpath. Each successive layer is placed on top of the previous one. The applied layers harden and join/sinter with adjacent layers, forming the desired 3D model.

Polyetheretherketone (PEEK) is a part of the range of polyaryletherketones (PAEKs) [10,11,12,13,14]. This polymer is resistant to high temperatures and chemical corrosion, has good strength, an increased modulus of elasticity and fracture toughness [15,16,17]. PEEK has also demonstrated excellent chemical stability and recyclability [18]. One of the fast developing areas of AM application is the production of prostheses, implants and other biomedical devices made of biocompatible polymers [19,20,21,22,23,24,25,26]. The biocompatibility, excellent fatigue and wear resistance of PEEK are well suited for such applications [27,28]. Thus, PEEK has a high potential in orthopedics [27,29,30] and traumatology [31] because it is a suitable candidate for replacing the metal components of implants [32,33,34,35,36].

The main challenges of 3D printing with PEEK are related to its high melting temperature (some 390 °C) and viscous behavior. The former leads to higher demands on printing equipment, while the latter prevents melting and additionally affects the strength of the molded parts because the process temperature drops rapidly after extrusion. A number of studies reported that manufacturing parameters, such as nozzle and table temperatures, layer thickness, printing speed, infill factor and infill angle, had an impact on the mechanical properties and appearance of finished PEEK products [37,38,39,40,41]. Wu et al. [42] investigated the influence of the layer thickness and the infill angle on the mechanical properties of printed PEEK parts. They found that optimal mechanical properties were achieved in samples with a layer thickness of 0.3 mm printed with an infill angle of 0°/90°. Deng et al. [43] studied the effect of printing speed, layer thickness, printing temperature and filling factor on the resulting tensile properties. The highest tensile properties of PEEK specimens were obtained at a printing speed of 60 mm/s, a layer thickness of 0.2 mm, a printing temperature of 370 °C and an infill factor of 40%. As reported by Arif et al. [22], the filament’s infill angle played an important role in the resulting mechanical properties of the samples. Zhao et al. [44] confirmed that the properties of samples could be improved by subsequent heat treatment. Despite a growing number of studies in this area, the optimal combination of 3D printing parameters for PEEK is still to be found, especially for biomedical applications, as they usually require fine printed structures with a small characteristic scale size. The reported results demonstrating better AM outcomes thanks to heat postprocessing could be a solution for achieving the desirable mechanical properties even for small-scale devices.

The different configurations of 3D-printed PEEK samples were studied. Tensile tests were carried out to determine the modulus of the elasticity of standard samples with different infill angles. Samples of as-delivered and extruded filament were also tested to assess their tensile strength. Compact tension (CT) samples were tested to determine fracture toughness (critical stress intensity factor) $K_{IC}$ and critical strain energy release rate $G_{IC}$. The changes in the properties of samples before and after heat treatment were analyzed.

## 2. Methods

### 2.1. Manufacturing of Samples

The samples were manufactured on an F2 Lite printer (F2 innovations, Russia). A PEEK filament with a diameter of 1.75 mm (REC, Russia) was extruded through 0.4 mm and 0.6 mm nozzles. To remove accumulated moisture, filaments were pre-dried at 120 °C for 24 h. A group of the samples were subjected to heat treatment after manufacturing: they were placed in quartz sand for uniform heating and baked at 220 °C for 72 h, followed by cooling to room temperature. According to the manufacturer’s statements, the material’s glass transition temperature is approximately 200 °C. The crystallization takes place in the range from the glass transition temperature to the melting temperature (343 °C); from this range, the heat treatment temperature was taken as 220 °C [45,46]. The baking time was chosen so that 1.5 h were required for the heat treatment of every millimeter of wall thickness. A Binder FP53 temperature chamber (BINDER GmbH, Tuttlingen, Germany) was used for the heat treatment of samples and the drying of filaments.

IdeaMaker software was used to determine a slicing sequence and a printing toolpath for a 3D printer. Before the manufacture, a special adhesive (Erich Krause^®^ Extra glue stick) was applied to the surface of the table. The contour surrounding the part was added. To hold its edges, helping to improve adhesion and prevent deformation. After manufacturing, the chamber was cooled to the room’s temperature by means of natural convection, and the parts were removed from the table. The contour structure surrounding the sample was manually detached. The FFF process parameters used in this study for the filament infill angles α = 45°, 90° and 180° when printing with a 0.6 mm nozzle and an infill angle α = 180° when printing with a 0.4 mm nozzle are given in Table 1.

The same parameters were used for CT samples, except for the layer height (0.2 mm) and the extrusion width (0.4 mm).

**Figure 1:**
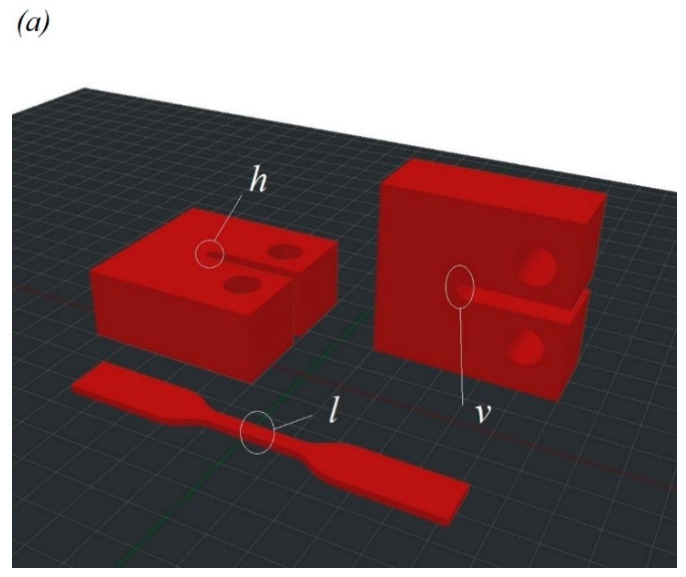

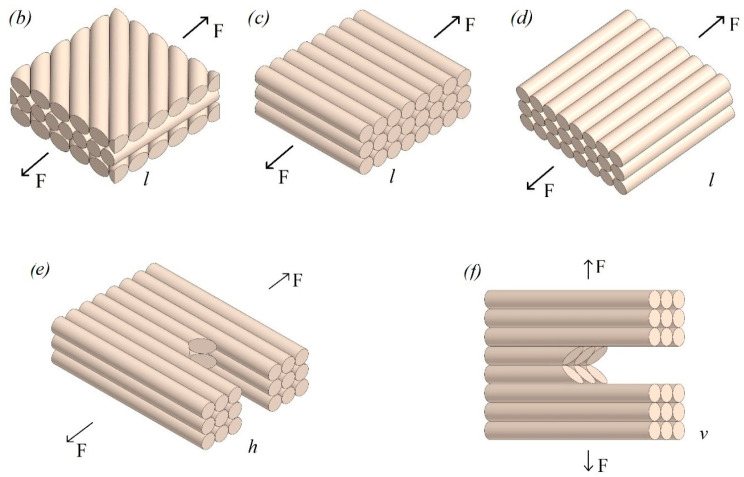
(**a**) Geometry of FFF manufactured PEEK samples for tensile and CT tests. Printing scheme for tensile samples for three configurations of infill angle: (**b**) α=45°, (**c**) α= 90° and (**d**) α= 180°. Configuration of the CT samples: (**e**) horizontal orientation *h*, (**f**) vertical orientation *v*.

**Figure 2:**
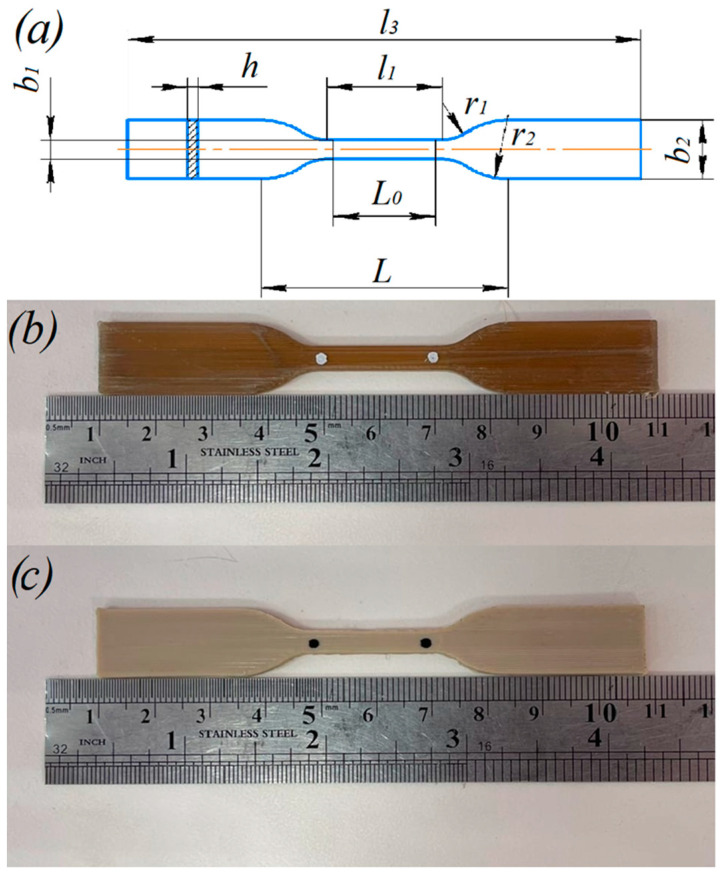
(**a**) Geometry of standard tensile test sample. PEEK sample without (**b**) and with (**c**) heat treatment.

The sample models used in this study are shown in Figure 1. As a well-known fact, the temperature gradient between the layers increases with the growth in the number of samples on the table due to the time required for the nozzle movement, leading to weak adhesion between the layers. To avoid this, the samples were printed one at a time in a single printing cycle. The dimensions and images of PEEK tensile samples according to ISO 527-2 are shown in Figure 2.

**Figure 3:**
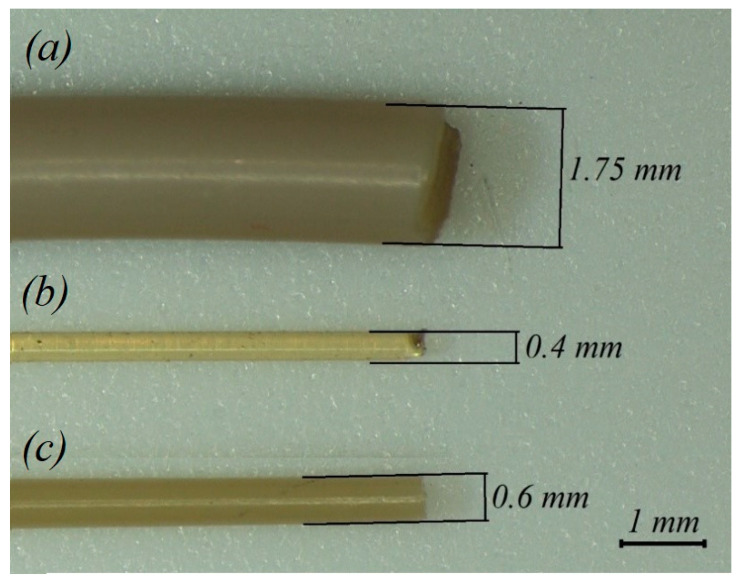
(**a**) As-delivered filament from coil; (**b**) filament extruded via 0.4 mm nozzle before heat treatment; and (**c**) filament extruded via 0.6 mm nozzle after heat treatment.

Samples of single-thread filament with a length of 85 mm extruded through nozzles with 0.4 mm and 0.6 mm diameter were also studied. Some of these samples were also subjected to heat treatment with the same conditions as beforementioned tensile samples. In addition, samples of as-delivered filament (before 3D printing) with the same length of 85 mm were prepared by cutting the filament from the coil (see Figure 3).

The samples with and without heat treatment had different colors: those without heat treatment were brown, indicating incomplete crystallization of the material. Heat treatment improved the interfacial adhesion between the layers, and such samples had a paler beige color (see Figure 2 and Figure 3). The main reason for incomplete crystallization is most often related to temperature fluctuations during the printing process.

### 2.2. Tensile Testing

Tensile tests were carried out in accordance with the ISO 527-2 standard on a Instron 68SC-5 universal testing machine with a 5 kN load cell at a constant displacement speed of 1 mm/min at room temperature (~22 °C). The accuracy of the load measurement in the range from 5 N to 5 kN was 0.5% of the measured value, and the resolution of the servo drive movement was 0.0095 µm. An AVE2 video extensometer was used to measure deformations. Two equidistant from the middle points located on the same axis were marked on the surface of the samples. The elongation of the test sample was measured via tracking the movement of these points by comparing and analyzing digital images during sample loading. The mechanical properties of the samples under tension were obtained from the graph of the dependence of stresses on digital deformation. The ability of specimens to absorb energy during deformation until fracture was also evaluated: the resilience and toughness moduli were calculated by the integration of the stress–strain curve from zero to the elastic limit and to the point of failure, respectively. To assess repeatability, each sample configuration was tested at least three times for samples without heat treatment and at least five times for samples in the case of heat treatment.

### 2.3. Fracture Tests

**Figure 4:**
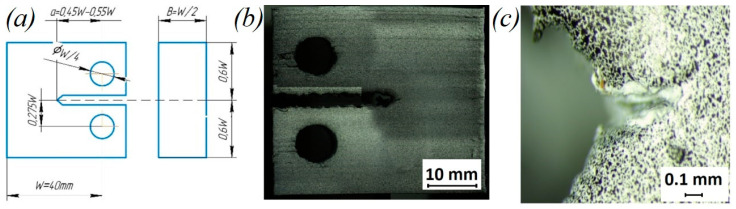
(**a**) Geometry of CT sample; (**b**) PEEK CT sample with speckles; and (**c**) enlarged view of crack is sharp edge area of sample.

The tensile fracture properties of the samples were evaluated in accordance with the ASTM D5045-14 standard for measuring the fracture toughness of polymers under plane deformation. CT specimens were manufactured in the configuration, as shown in Figure 4a, with W = 40 mm. The supports in the crack zone were removed after printing using a desktop drilling and milling machine. In accordance with the ASTM D5045 standard, the crack tip in samples with horizontal and vertical orientations was made with a sharp 0.08 mm thick razor blade. Before the test, the samples were kept in a drying chamber for 24 h at a temperature of 70 °C to remove moisture. The traverse speed of the Instron 68SC-5 universal testing machine was 1 mm/min. Assuming the linear elastic fracture, the critical stress intensity factor $K_{IC}$ was determined in accordance with the method given in ASTM D5045 by the following formula:

(1) $$K_{IC}=\frac{P_{Q}}{BW^{1/2}}f\left(x\right),$$

where $P_{Q}$ is the load, *B* is the sample thickness, *W* is the sample width, x = a/W, a is a crack length,

(2) $$f\left(x\right)=\frac{\left(2+x\right)\left(0.886+4.64x-13.32x^{2}+14.72x^{3}-5.6x^{4}\right)}{{\left(1-x\right)}^{\frac{3}{2}}}$$

The critical rate of strain energy release was also determined:

(3) $$G_{IC}=\frac{\left(1-\nu^{2}\right)K_{IC}{}^{2}}{E},$$

where *E* is the elasticity modulus measured in the tests, and ν is the Poisson’s ratio. 

To ensure the validity of the $K_{IC}$, the criterion described in Section 9.1.1 of ASTM D5045 was used. In addition, the sample size was selected in accordance with this criterion, which provided a plane-strain state at the crack tip. The Vic 3D Micro-DIC digital image correlation system was used to measure strain fields on a sample surface. For this, a random black-and-white pattern was applied to the samples using an airbrush. Sequential images were obtained using two 5.0-megapixel cameras to assess the displacements of speckles. The average deformation in the crack-opening zone was estimated using Vic 3D v9 software. The properties of the samples during fracture were obtained from the stress–strain curve. To assess repeatability, each sample configuration was tested at least three times.

### 2.4. Numerical Calculations

The finite-element (FE) method implemented in SIMULIA Abaqus software was used to model the mechanical behavior of CT samples. The finite-element models of the studied samples were created based on discretization with the linear hexahedral finite elements of C3D8R type. The properties of the real material were used in simulations: the elastic modulus E=4.7 GPa, and the Poisson’s ratio ν=0.38 [44].

Two reference points connected with the upper and lower surfaces of the holes in specimens were created for boundary conditions. The condition of rigid fixation was set for the lower reference point, with restrictions on all movements except for the vertical one applied to the upper one. All movements and rotations were prohibited, except for U2 and UR1, as restrictions for the whole model. The load equal to 4950 N was set as concentrated force applied to the upper reference point.

Calculations were implemented in the SIMULIA Abaqus Standard application package using the extended finite-element method (XFEM). The basic idea of this method is to add the enrichment of the approximation of a finite-element near a crack by discontinuous functions [47,48]. The crack location is described in XFEM using the level-set method. A function with zero values for the crack is introduced. Then, the observed region is divided into two subdomains, in which this function takes positive and negative values. XFEM-based crack modeling allows the prediction of both stationary and moving cracks [49]. In this paper, the linear-elastic fracture mechanics (LEFM) approach was chosen to model the moving cracks [50]. Crack initiation occurs when the adhesion of the enriched elements deteriorates. A crack appears inside the element when stresses or strains in this element meet certain criteria for crack initiation [51]. The criterion of maximum principal stresses was chosen as the failure criterion in this study.

## 3. Results and Discussion

### 3.1. Effect of Filament Infill Angle on Tensile Properties

**Figure 5:**
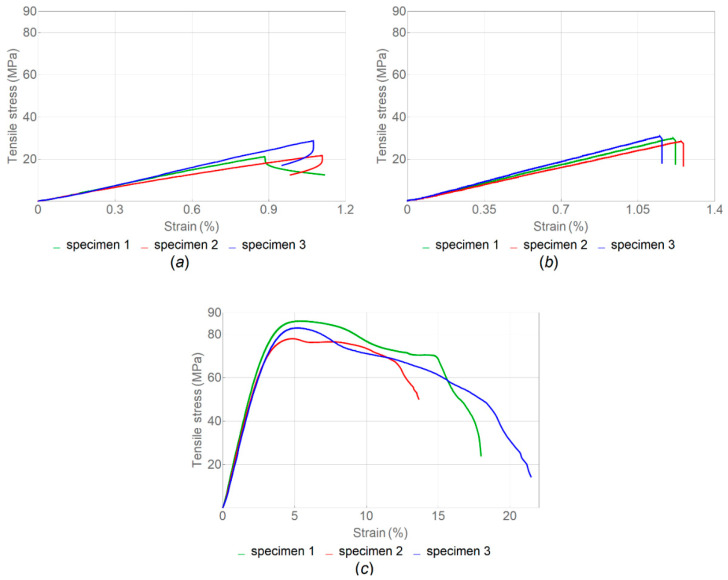
Stress–strain curve for tension load of samples without heat treatment with different infill angle α: (**a**) 45°; (**b**) 90°; and (**c**) 180° (nozzle diameter, 0.6 mm).

**Figure 6:**
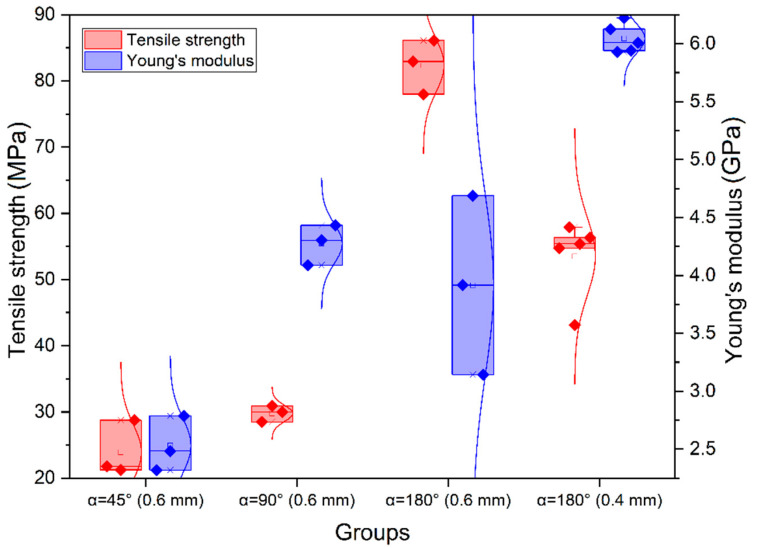
Results for tensile strength and elastic modulus of samples without heat treatment for α = 45°, α = 90° and α = 180° (nozzle diameter is given in brackets).

The tensile strength of samples manufactured with three different sets of parameters was evaluated. The stress–strain curves obtained in tensile tests of samples with infill angle α = 45°, α = 90° and α = 180° without heat treatment are shown in Figure 5; the corresponding boxplot is given in Figure 6.

The properties of samples without heat treatment obtained in the tensile tests are presented in Table 2. According to ISO 527-2, the tensile strength corresponded to the stress, at which the first local maximum was observed.

It was found that samples with α = 180° had the highest tensile strength on average, followed by samples with α = 90° and α = 45°, while the average value of the elastic modulus was higher for samples with α = 90°, followed by α = 180° and α = 45° (Table 2). Generally, the samples with α = 180° exhibited the highest values for all mechanical properties. This may be because the load was applied along the print layers. The same results were obtained by Arif et al. [16]. Unlike the samples with α = 45° and α = 90°, the samples with α = 180° demonstrated a zone of plastic deformation (Figure 6c). This was also confirmed by the value of the toughness modulus (Table 2), which was nearly two orders of magnitude higher for the last case.

**Figure 7:**
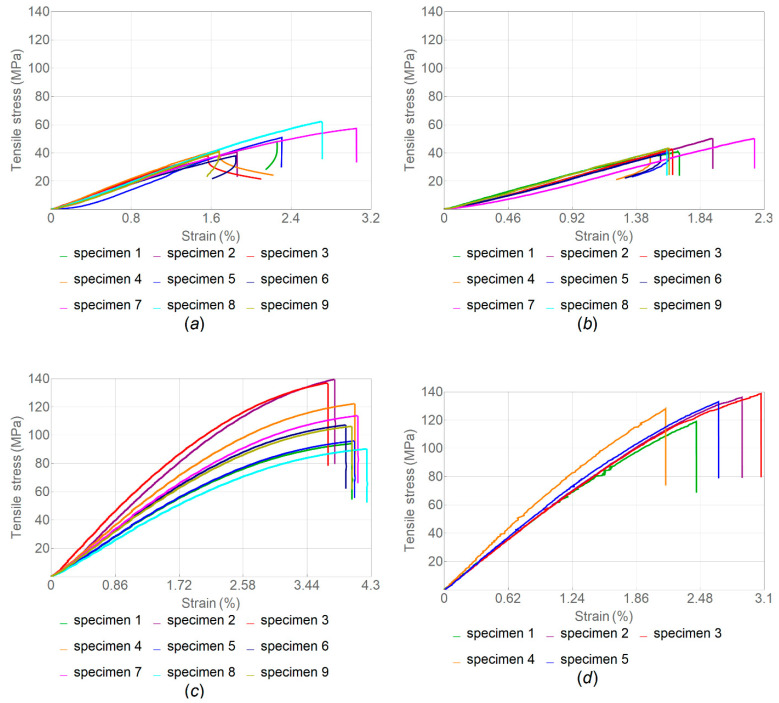
Tensile stress–strain curves for samples after heat treatment with different infill angle α: (**a**) 45°; (**b**) 90°; (**c**) 180°; and (**d**) 180° (nozzle diameter, 0.4 mm in (**d**) and 0.6 mm in (**a**–**c**)).

**Figure 8:**
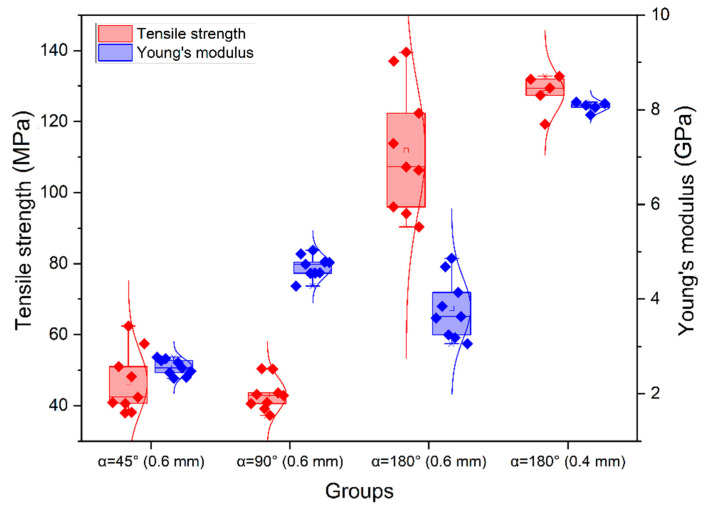
Results for tensile strength and elastic modulus of heat-treated samples for α = 45°, α = 90° and α = 180°.

The stress–strain curves for a series of tensile tests of samples with various infill angle after the heat treatment are given in Figure 7; the corresponding boxplot is shown in Figure 8. The overview of these results is given in Table 3.

The samples with α = 180° had the highest tensile strength on average, followed by samples with α = 45° and α = 90°; the average value of the elastic modulus was also higher for samples with α = 180°, followed by samples with α = 90° and α = 45°.

**Figure 9:**
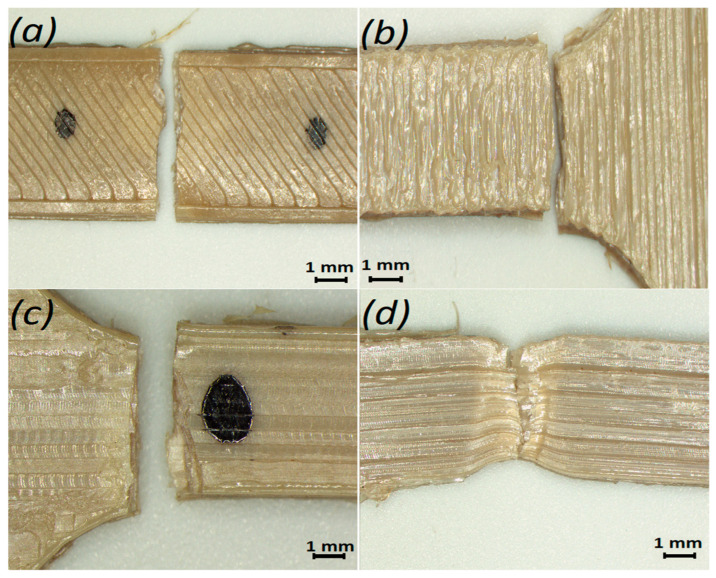
Fractured samples with different infill angles α: (**a**) 45°; (**b**) 90°; (**c**) 180° with heat treatment; and (**d**) 180° without heat treatment.

In contrast with the tests of samples without heat treatment, samples with the infill angle α = 180° demonstrated no plastic deformation zone, which indicates that they became more brittle after such treatment. This can be clearly seen when comparing the resilience and toughness values for samples with and without heat treatment. For samples with the infill angle α = 45°, the difference was 76.3% for resilience and 75.6% for toughness. For samples with the infill angle α = 90°, the respective differences were 50.4% and 49.6%, while for the infill angle α = 180°, they were 6.7% and 313.4%. Images of the fractured samples under tension are shown in Figure 9.

It was concluded that the modulus of elasticity of heat-treated samples increased by less than 1% for samples with α = 45°, by 9.2% for samples with α = 90° and by 19% for samples with α = 180°. It was also found that PEEK samples with α = 180° showed the highest tensile strength, while samples with α = 90°—the lowest. Such a low strength value was due to the geometry of filaments deposited in layers during FFF manufacturing, resulting in stress concentration [52]. It is obvious that the heat treatment resulted in an increase in the strength properties of the PEEK samples. The tensile strength for samples with α = 45° increased by 64%, for samples with α = 90° by 37% and for samples with α = 180° by 43%. Although the elastic modulus and tensile strength parameters are higher in heat-treated specimens with the infill angle α = 180°, the resilience and toughness values, which demonstrate the ability of the material to absorb energy during elastic and plastic deformation up to the point of failure, are higher in non-treated specimens (Table 2 and Table 3). In addition, Figure 7 and Table 3 show that the tensile strength of all heat-treated samples was several times higher than that of samples without heat treatment. 

Because the samples with α = 180° showed higher mechanical properties, another batch of samples was produced with the same printing parameters but using a nozzle with a diameter of 0.4 mm. The obtained results are presented in Table 3 and the respective stress–strain curves in Figure 7d. The values obtained for the ultimate strength of samples without heat treatment printed with a 0.4 mm nozzle differ from those for samples printed with a 0.6 mm nozzle—down by 42%—while the modulus of elasticity increased by 43%. For the heat-treated samples, the tensile strength varied by less than 1%, and the modulus of elasticity increased by 52% thanks to the larger number of tool passes in one layer: there were 6 passes for a 0.6 mm nozzle and 10 passes for a 0.4 mm nozzle.

### 3.2. Investigation of Filament Properties

**Figure 10:**
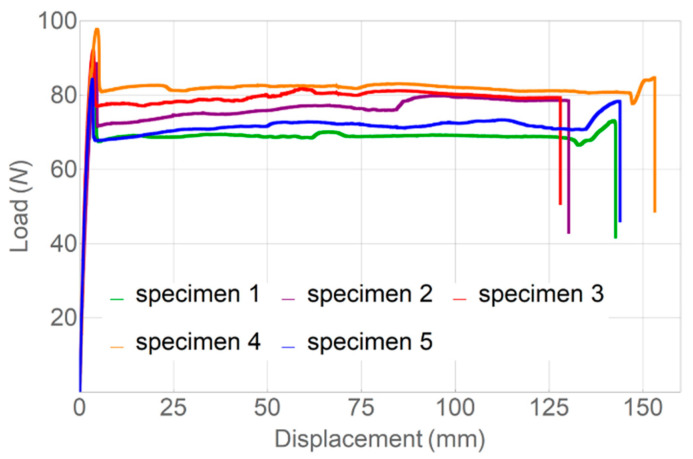
Stress–strain diagrams for as-delivered filament.

**Figure 11:**
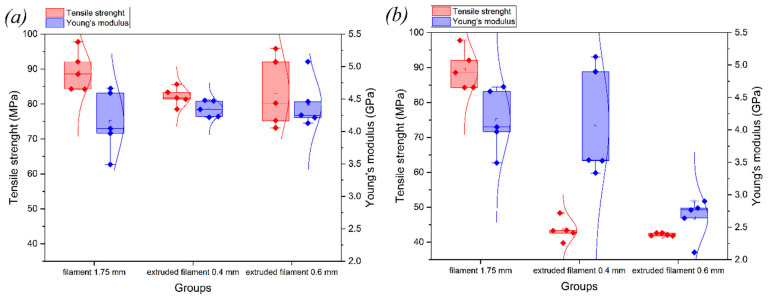
Results for ultimate strength and modulus of elasticity from tensile tests for as-delivered and extruded filament: (**a**) with heat treatment and (**b**) without heat treatment.

Tests were performed to assess the tensile strength of the original as-delivered filament, as well as the single-thread printed samples. The respective stress–strain curves for the tensile tests of as-delivered filament are given in Figure 10 with the corresponding boxplot in Figure 11.

On the basis of the obtained data, it was concluded that the pure filament in terms of tensile strength and the modulus of elasticity is similar to the samples printed with the 0.6 mm nozzle without the use of the subsequent heat treatment. These values are found to be within a 10% range (see Table 4).

**Figure 12:**
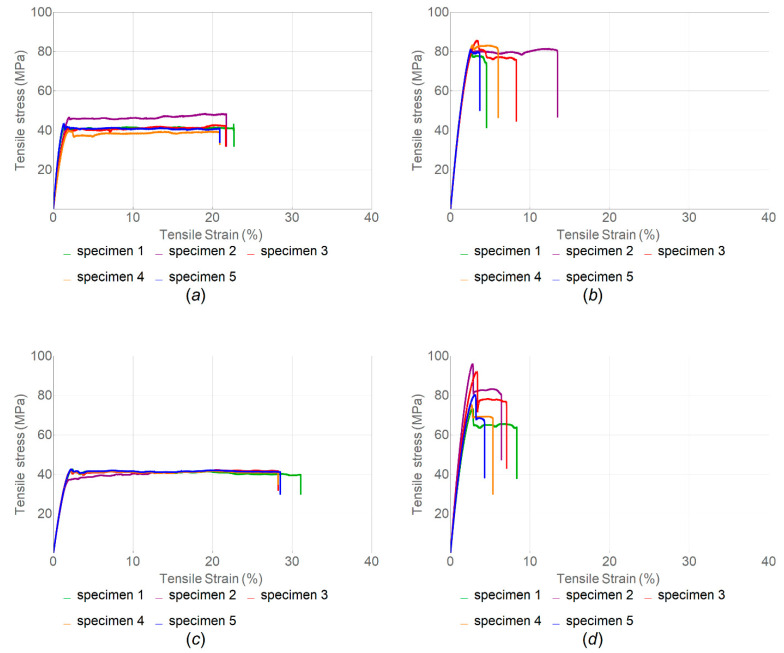
Stress–strain diagrams for extruded filament printed with (**b**,**d**) and without (**a**,**c**) heat treatment, ((**a**,**b**)—0.4 mm nozzle; (**c**,**d**)—0.6 mm nozzle).

In addition, the filaments extruded through the nozzles with diameters of 0.4 mm and 0.6 mm with and without heat treatment were tested under tensile loading. The obtained results are presented in Table 5, while the stress–strain curves for the extruded filament with and without heat treatment (Figure 3) are shown in Figure 12.

The graphs in Figure 12 demonstrate that the plastic deformation zone decreased for all single-thread filament samples subjected to heat treatment. When comparing resilience values, the difference between its magnitude for the extruded filament without and with heat treatment was 68.1% (0.4 mm nozzle) and 60.4% (0.6 mm nozzle), while for toughness it was 78.3% and 194.4%, respectively. During the tensile test, the cross-section of the filament decreased from the initial size to 0.2–0.25 mm. The values of the elastic modulus of the as-delivered filament in comparison with an extruded one without the heat treatment exhibited a difference of 2%; it was within 7% compared to that of an extruded filament after the heat treatment. In addition, the elastic modulus for the filament without the subsequent heat treatment extruded with nozzles of different diameters showed a difference of 1%, with a higher value for the filament extruded with the 0.6 mm nozzle. For the filament subjected to the subsequent heat treatment, the difference varied within 2%, also with a higher value for the sample extruded with the 0.6 mm nozzle. In terms of tensile strength, the as-delivered filament exceeded the one extruded through nozzles with diameters of 0.4 mm and 0.6 mm using the heat treatment by 9% and 7%, respectively, and without the heat treatment by 69% and 72%, respectively. The differences in the values may be due to filament manufacturing issues and also the fact that its diameter is larger than the diameter of the filament extruded through the nozzle. It was found that the modulus of elasticity was within the same limits as that of the samples subjected to the heat treatment after printing, but the tensile strength of the finished products was greater in the case of the orientation of the layers along the direction of the load.

### 3.3. Critical Stress Intensity Factor and Critical Strain Energy Release Rate

**Figure 13:**
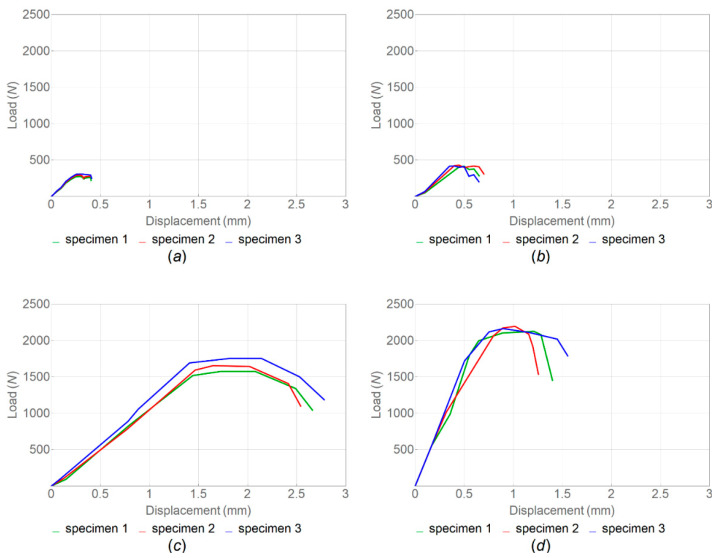
Load–displacement curves for PEEK samples printed with vertical (**a**,**b**) and horizontal (**c**,**d**) orientations ((**a**,**c**)—no heat treatment; (**b**,**d**)—with heat treatment; and infill angle—90°).

**Figure 14:**
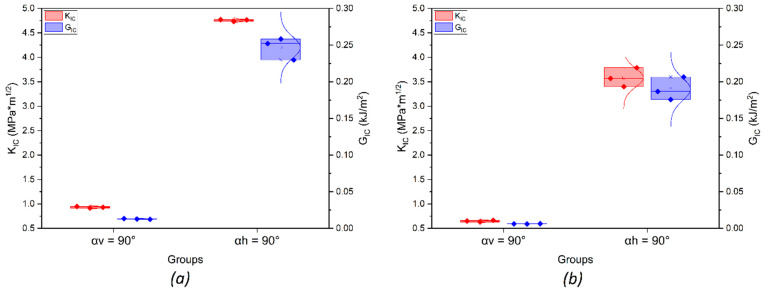
Results for stress intensity factor and critical strain energy release rate for samples with (**a**) and without (**b**) heat treatment.

The fracture-test results demonstrated the effect of FFF manufacturing parameters and the infill angle orientation on fracture toughness. Poisson’s ratio was assumed to be 0.38, following [44]. As can be seen from the load–displacement curves (Figure 13) and the calculated values of *K_IC_* and *G_IC_* (see Table 6 and Figure 14), the samples printed with horizontal orientation showed significantly better characteristics. The subsequent heat treatment increased the fracture resistance by 33% for samples with horizontal orientation and by 45% for samples with vertical orientation. The results demonstrate that the orientation of the layers with respect to the direction of the load has the greatest effect on the mechanical characteristics. The difference between the samples with horizontal and vertical orientations was associated with different filament-scale geometrical features, as the printing areas for these samples were different. Additionally, in the samples with vertical orientation, the layers were overlapped faster during manufacturing because the sample was placed vertically, and there was not enough time for the layers to cool down completely. After reaching the maximum load, softening was observed due to the deviation of the crack into the adjacent interlayer boundaries (Figure 14c,d).

**Figure 15:**
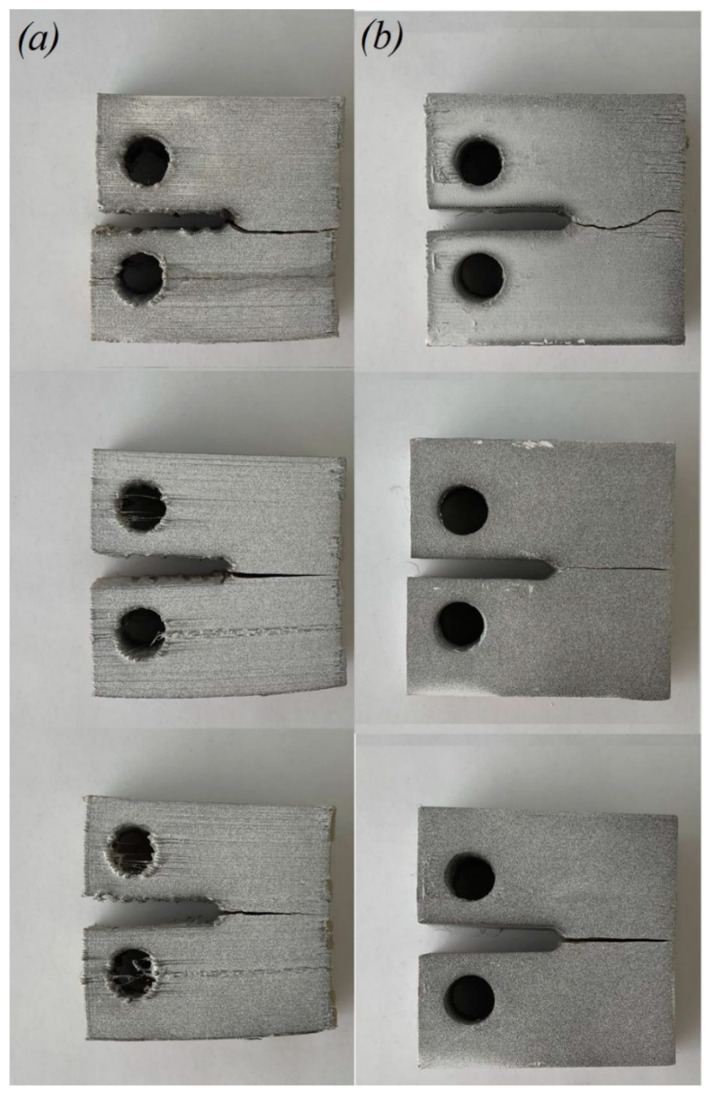
Fractured CT samples printed with vertical (**a**) and horizontal (**b**) orientations.

Images of the fracture surfaces of CT samples after tension are shown in Figure 15. The crack tip in the samples with horizontal and vertical orientations during compact tension showed a higher probability of moving through the layer interface, where the interfacial zone between the layers acted as the weakest link. The crack growth after the maximum load was observed at a constant applied tensile velocity in a sample with vertical orientation due to the presence of voids at the layer interfaces. These voids constrained the growth of the crack in this direction. The fracture, in this case, was characterized by oscillatory velocities of the crack tip and crack growth jumps, leading to periodic load fluctuations (Figure 13a,b).

**Figure 16:**
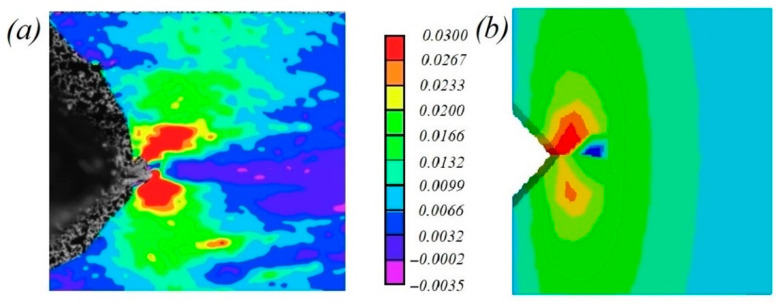
(**a**) Axial strains obtained using micro-DIC for heat-treated sample with horizontal orientation at maximum load value; (**b**) FE analysis performed in Abaqus CAE for same load.

**Figure 17:**
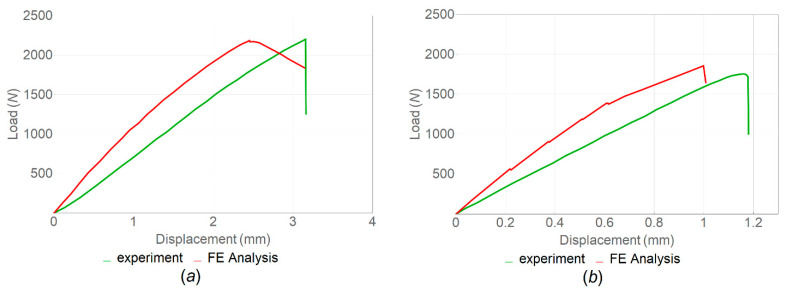
Load–displacements curves obtained from FE analysis and the experiments for samples with (**a**) and without (**b**) heat treatment.

The strain fields obtained at the maximum load value in the experiment using a digital image correlation system were evaluated and compared with the results of numerical simulations. It was revealed that the deformation was maximal at the crack front (Figure 16). The load–displacements curves were compared for the experimentally and numerically calculated mean values (Figure 17); the highest value of the maximum strain was found in the samples with a horizontal orientation.

It was found that the tensile strength of CT samples after heat treatment with horizontal and vertical orientation was 2.19 MPa and 0.32 MPa, respectively, while the difference in fracture toughness values between them was 79.6% (see Table 6). The low fracture toughness for the samples with vertical orientation reduced the full potential of PEEK for high-load applications, but its value was still within the range of brittle polymers such as acrylic, epoxy resin and polystyrene.

## 4. Conclusions

The tensile properties of PEEK samples manufactured using the FFF technique with nozzles of different diameters were studied. Three sample configurations with infill angles α = 45°, 90° and 180° were tested in accordance with the ISO 527-2 standard. It was established that the postprocessing heat treatment of the manufactured samples contributed to the interfacial strength of the layers, which, in turn, demonstrated a significant effect on the tensile strength properties. Samples with α = 180° showed the highest levels of tensile strength and modulus of elasticity, followed by filling at an angle of α = 90° and α = 45°.

Heat treatment also improved the properties for CT samples. Weak interfacial strength resulted in a crack deviation into the adjacent interlayer boundaries after reaching the maximum load. The fracture was characterized by oscillatory velocities of the crack tip and growth jumps, leading to periodic fluctuations in the load value.

The tensile strength of the single filament samples of various configurations was estimated. In terms of tensile strength, the pure filament exceeds the filament extruded through nozzles with a diameter of 0.4 mm and 0.6 mm with the use of heat treatment by 9% and 7%, respectively, and without the heat treatment by 69% and 72%, respectively, demonstrating the importance of post-manufacture treatment. The modulus of elasticity lies within the same limits as for the samples subjected to the heat treatment after printing, but the tensile strength of the finished products was greater for the orientation of the layers along the direction of the load.

## Figures and Tables

**Table 1:** Manufacturing parameters.

Process Parameter	Value
Nozzle movement speed, mm/min	2100
Nozzle temperature, °C	435
Table temperature, °C	145
Chamber temperature, °C	75
Layer height, mm	0.1
Extrusion width, mm	0.4–0.6
Infill	Straight-line
Infill density, %	100
Airflow, %	100

**Table 2:** Tensile properties of samples without heat treatment for α = 45°, α = 90° and α = 180° (nozzle diameter, 0.6 mm).

	α = 45°	α = 90°	α = 180°
Elastic modulus, E (GPa)	2.53 ± 0.23	4.27 ± 0.17	3.92 ± 0.77
Tensile strength, $\sigma_{s}\;\;$(MPa)	23.91 ± 4.18	29.77 ± 1.21	82.34 ± 4.08
Resilience (J·m^−3^)	12.59 ± 2.83	17.90 ± 0.23	288.93 ± 42.88
Toughness (J·m^−3^)	13.73 ± 1.35	18.19 ± 0.23	1121.16 ± 210.30

**Table 3:** Tensile properties of samples with heat treatment and filament infill angle α = 45°, α = 90° and α = 180°.

	Nozzle 0.6 mm			Nozzle 0.4 mm
	α = 45	α = 90°	α = 180°	α = 180°
Elastic modulus, E (GPa)	2.53 ± 0.15	4.68 ± 0.23	4.74 ± 0.12	8.06 ± 0.11
Tensile strength, $\sigma_{s}\;$(MPa)	46.53 ± 8.81	43.11 ± 4.58	127.76 ± 6	128.15 ± 5.41
Resilience (J·m^−3^)	53.13 ± 24.88	36.06 ± 8.45	270.78 ± 32.79	202.02 ± 40.46
Toughness (J·m^−3^)	56.27 ± 23.07	36.10 ± 8.45	271.22 ± 33.00	202.19 ± 40.47

**Table 4:** Tensile properties of as-delivered filament samples in comparison with standard printed samples without heat treatment.

	As-Delivered Filament	Standard Printed Samples without Heat Treatment		
		α = 45°	α = 90°	α = 180°
Elastic modulus, E (GPa)	4.15 ± 0.48	2.53 ± 0.23	4.27 ± 0.17	3.92 ± 0.77
Tensile strength, $\sigma_{s}\;$(MPa)	89.39 ± 5.71	23.91 ± 4.18	29.77 ± 1.21	82.34 ± 4.08
Resilience (J·m^−3^)	231.74 ± 42.90	12.59 ± 2.83	17.90 ± 0.23	288.93 ± 42.88
Toughness (J·m^−3^)	10,508.89 ± 1117.59	13.73 ± 1.35	18.19 ± 0.23	1121.16 ± 210.30

**Table 5:** Tensile properties of samples of filament extruded with nozzles (0.4 mm and 0.6 mm), with and without heat treatment.

	Without Heat Treatment		With Heat Treatment	
	Nozzle 0.4 mm	Nozzle 0.6 mm	Nozzle 0.4 mm	Nozzle 0.6 mm
Elastic modulus, E (GPa)	4.08 ± 0.85	4.12 ± 0.31	4.35 ± 0.12	4.43 ± 0.38
Tensile strength, $\sigma_{s}\;$(MPa)	43.51 ± 3.11	42.21 ± 0.41	82.08 ± 2.62	83.32 ± 10.16
Resilience (J·m^−3^)	44.53 ± 9.09	59.19 ± 1.94	139.55 ± 25.82	149.53 ± 28.60
Toughness (J·m^−3^)	872.48 ± 76.02	1160.06 ± 43.14	489.27 ± 312.46	394.01 ± 113.98

**Table 6:** Critical stress intensity factor, critical strain energy release rate and tensile strength obtained in CT tests.

	Samples without Heat Treatment		Samples with Heat Treatment	
	Vertical Orientation	Horizontal Orientation	Vertical Orientation	Horizontal Orientation
Critical stress intensity factor, $K_{IC}\;$ ($\mathrm{MPa}\cdot m^{1/2}$)	0.64 ± 0.02	3.58 ± 0.20	0.93 ± 0.02	4.76 ± 0.02
Critical strain energy release rate, $G_{IC}\;$ (kJ/$m^{2}$)	0.01 ± 0.01	0.21 ± 0.02	0.02 ± 0.01	0.25 ± 0.02
Tensile strength, $\sigma_{s}$ (MPa)	0.22 ± 0.01	1.66 ± 0.10	0.33 ± 0.02	2.16 ± 0.03

## Data Availability

The data presented in this study are available on request from the corresponding author.

## References

[1] Plocher J., Panesar A. (2019). Review on Design and Structural Optimisation in Additive Manufacturing: Towards next-Generation Lightweight Structures. Mater. Des..

[2] Guo N., Leu M.C. (2013). Additive Manufacturing: Technology, Applications and Research Needs. Front. Mech. Eng..

[3] Kumar S., Wardle B.L., Arif M.F. (2017). Strength and Performance Enhancement of Bonded Joints by Spatial Tailoring of Adhesive Compliance via 3D Printing. ACS Appl. Mater. Interfaces.

[4] Kumar S., Wardle B.L., Arif M.F., Ubaid J. (2018). Stress Reduction of 3D Printed Compliance-Tailored Multilayers. Adv. Eng. Mater..

[5] Liljenhjerte J., Kumar S. (2015). Pull-out Performance of 3D Printed Composites with Embedded Fins on the Fiber. Mater. Res. Soc..

[6] Liljenhjerte J., Upadhyaya P., Kumar S. (2016). Hyperelastic Strain Measurements and Constitutive Parameters Identification of 3D Printed Soft Polymers by Image Processing. Addit. Manuf..

[7] Rajan K., Samykano M., Kadirgama K., Harun W.S.W., Rahman M.M. (2022). Fused Deposition Modeling: Process, Materials, Parameters, Properties, and Applications. Int. J. Adv. Manuf. Technol..

[8] Valino A.D., Dizon J.R.C., Espera A.H., Chen Q., Messman J., Advincula R.C. (2019). Advances in 3D Printing of Thermoplastic Polymer Composites and Nanocomposites. Prog. Polym. Sci..

[9] Das A., Marnot A.E.C., Fallon J.J., Martin S.M., Joseph E.G., Bortner M.J. (2020). Material Extrusion-Based Additive Manufacturing with Blends of Polypropylene and Hydrocarbon Resins. ACS Appl. Polym. Mater..

[10] Kurtz S.M. (2012). An Overview of PEEK Biomaterials.

[11] Mark H.F. (2001). Acetylenic Polymers, Substituted. Encyclopedia of Polymer Science and Technology.

[12] Wang Y., Müller W.D., Rumjahn A., Schmidt F., Schwitalla A.D. (2021). Mechanical Properties of Fused Filament Fabricated PEEK for Biomedical Applications Depending on Additive Manufacturing Parameters. J. Mech. Behav. Biomed. Mater..

[13] Dua R., Rashad Z., Spears J., Dunn G., Maxwell M. (2021). Applications of 3d-Printed Peek via Fused Filament Fabrication: A Systematic Review. Polymers.

[14] Zanjanijam A.R., Major I., Lyons J.G., Lafont U., Devine D.M. (2020). Fused Filament Fabrication of Peek: A Review of Process-Structure-Property Relationships. Polymers.

[15] Sharma G., Vuppuluri A., Suresh K. (2022). Essential Work of Fracture Studies of 3D Printed PEEK (Poly-Ether-Ether-Ketone) Polymer. Eng. Fract. Mech..

[16] Hart K.R., Dunn R.M., Sietins J.M., Hofmeister Mock C.M., Mackay M.E., Wetzel E.D. (2018). Increased Fracture Toughness of Additively Manufactured Amorphous Thermoplastics via Thermal Annealing. Polymer.

[17] Hart K.R., Dunn R.M., Wetzel E.D. (2020). Increased Fracture Toughness of Additively Manufactured Semi-Crystalline Thermoplastics via Thermal Annealing. Polymer.

[18] McLauchlin A.R., Ghita O.R., Savage L. (2014). Studies on the Reprocessability of Poly(Ether Ether Ketone) (PEEK). J. Mater. Process. Technol..

[19] Javaid M., Haleem A. (2018). Additive Manufacturing Applications in Medical Cases: A Literature Based Review. Alex. J. Med..

[20] Lee N. (2016). The Lancet Technology: 3D Printing for Instruments, Models, and Organs?. Lancet.

[21] Lupuleasa D., Drăgănescu D., Hîncu L., Tudosă C.P., Cioacă D. (2018). Biocompatible Polymers for 3D Printing. Farmacia.

[22] Arif M.F., Kumar S., Varadarajan K.M., Cantwell W.J. (2018). Performance of Biocompatible PEEK Processed by Fused Deposition Additive Manufacturing. Mater. Des..

[23] Singh D., Singh R., Boparai K.S. (2018). Development and Surface Improvement of FDM Pattern Based Investment Casting of Biomedical Implants: A State of Art Review. J. Manuf. Process..

[24] Karimipour-Fard P., Behravesh A.H., Jones-Taggart H., Pop-Iliev R., Rizvi G. (2020). Effects of Design, Porosity and Biodegradation on Mechanical and Morphological Properties of Additive-Manufactured Triply Periodic Minimal Surface Scaffolds. J. Mech. Behav. Biomed. Mater..

[25] Germain L., Fuentes C.A., van Vuure A.W., des Rieux A., Dupont-Gillain C. (2018). 3D-Printed Biodegradable Gyroid Scaffolds for Tissue Engineering Applications. Mater. Des..

[26] Singh S., Prakash C., Ramakrishna S. (2019). 3D Printing of Polyether-Ether-Ketone for Biomedical Applications. Eur. Polym. J..

[27] Kurtz S.M., Devine J.N. (2007). PEEK Biomaterials in Trauma, Orthopedic, and Spinal Implants. Biomaterials.

[28] Das A., Chatham C.A., Fallon J.J., Zawaski C.E., Gilmer E.L., Williams C.B., Bortner M.J. (2020). Current Understanding and Challenges in High Temperature Additive Manufacturing of Engineering Thermoplastic Polymers. Addit. Manuf..

[29] Liao K. (1994). Performance Characterization and Modeling of a Composite Hip Prosthesis. Exp. Tech..

[30] Gao S., Liu R., Xin H., Liang H., Wang Y., Jia J. (2021). The Surface Characteristics, Microstructure and Mechanical Properties of Peek Printed by Fused Deposition Modeling with Different Raster Angles. Polymer.

[31] Kelsey D.J., Springer G.S., Goodman S.B. (1997). Composite Implant for Bone Replacement. J. Compos. Mater..

[32] Sicilia A., Cuesta S., Coma G., Arregui I., Guisasola C., Ruiz E., Maestro A. (2008). Titanium Allergy in Dental Implant Patients: A Clinical Study on 1500 Consecutive Patients. Clin. Oral Implant. Res..

[33] Ma H., Suonan A., Zhou J., Yuan Q., Liu L., Zhao X., Lou X., Yang C., Li D., Zhang Y. (2021). gang PEEK (Polyether-Ether-Ketone) and Its Composite Materials in Orthopedic Implantation. Arab. J. Chem..

[34] Lommen J., Schorn L., Sproll C., Haussmann J., Kübler N.R., Budach W., Rana M., Tamaskovics B. (2022). Reduction of CT Artifacts Using Polyetheretherketone (PEEK), Polyetherketoneketone (PEKK), Polyphenylsulfone (PPSU), and Polyethylene (PE) Reconstruction Plates in Oral Oncology. J. Oral Maxillofac. Surg..

[35] Jiang C.-P., Cheng Y.-C., Lin H.-W., Chang Y.-L., Pasang T., Lee S.-Y. (2022). Optimization of FDM 3D Printing Parameters for High Strength PEEK Using the Taguchi Method and Experimental Validation. Rapid Prototyp. J..

[36] Yang C., Tian X., Li D., Cao Y., Zhao F., Shi C. (2017). Influence of Thermal Processing Conditions in 3D Printing on the Crystallinity and Mechanical Properties of PEEK Material. J. Mater. Process. Technol..

[37] Wang Y., Müller W.D., Rumjahn A., Schwitalla A. (2020). Parameters Influencing the Outcome of Additive Manufacturing of Tiny Medical Devices Based on PEEK. Materials.

[38] Challa B.T., Gummadi S.K., Elhattab K., Ahlstrom J., Sikder P. (2022). In-House Processing of 3D Printable Polyetheretherketone (PEEK) Filaments and the Effect of Fused Deposition Modeling Parameters on 3D-Printed PEEK Structures. Int. J. Adv. Manuf. Technol..

[39] Akhoundi B., Nabipour M., Hajami F., Shakoori D. (2020). An Experimental Study of Nozzle Temperature and Heat Treatment (Annealing) Effects on Mechanical Properties of High-Temperature Polylactic Acid in Fused Deposition Modeling. Polym. Eng. Sci..

[40] Fitzharris E.R., Watt I., Rosen D.W., Shofner M.L. (2018). Interlayer Bonding Improvement of Material Extrusion Parts with Polyphenylene Sulfide Using the Taguchi Method. Addit. Manuf..

[41] Liaw C.Y., Tolbert J.W., Chow L.W., Guvendiren M. (2021). Interlayer Bonding Strength of 3D Printed PEEK Specimens. Soft Matter.

[42] Wu W., Geng P., Li G., Zhao D., Zhang H., Zhao J. (2015). Influence of Layer Thickness and Raster Angle on the Mechanical Properties of 3D-Printed PEEK and a Comparative Mechanical Study between PEEK and ABS. Materials.

[43] Deng X., Zeng Z., Peng B., Yan S., Ke W. (2018). Mechanical Properties Optimization of Poly-Ether-Ether-Ketone via Fused Deposition Modeling. Materials.

[44] Zhao Y., Zhao K., Li Y., Chen F. (2020). Mechanical Characterization of Biocompatible PEEK by FDM. J. Manuf. Process..

[45] Tardif X., Pignon B., Boyard N., Schmelzer J.W.P., Sobotka V., Delaunay D., Schick C. (2014). Experimental Study of Crystallization of PolyEtherEtherKetone (PEEK) over a Large Temperature Range Using a Nano-Calorimeter. Polym. Test..

[46] Balani S.B. (2019). Additive Manufacturing of the High-Performance Thermoplastics: Experimental Study and Numerical Simulation of the Fused Filament Fabricatio. Ph.D. Thesis.

[47] Abdelaziz Y., Hamouine A. (2008). A Survey of the Extended Finite Element. Comput. Struct..

[48] Belytschko T., Black T. (1999). Elastic Crack Growth in Finite Elements with Minimal Remeshing. Int. J. Numer. Methods Eng..

[49] Zhang C., Cao P., Cao Y., Li J. (2013). Using Finite Element Software to Simulation Fracture Behavior of Three-Point Bending Beam with Initial Crack. J. Softw..

[50] Du Z. (2016). Extended Finite Element Method (XFEM) in Abaqus Dassault System.

[51] Remmers J.J.C., de Borst R., Needleman A. (2008). The Simulation of Dynamic Crack Propagation Using the Cohesive Segments Method. J. Mech. Phys. Solids.

[52] Allum J., Gleadall A., Silberschmidt V.V. (2020). Fracture of 3D-Printed Polymers: Crucial Role of Filament-Scale Geometric Features. Eng. Fract. Mech..

